# Mechanistic insights and biomarker discovery in immune cell aging and age-associated diseases

**DOI:** 10.3389/fimmu.2025.1637191

**Published:** 2025-10-08

**Authors:** Prabhat Upadhyay, Aamir Suhail, Pukar Khanal, Sudhir Kumar

**Affiliations:** ^1^ Vincent Center for Reproductive Biology, Department of Ob/Gyn, Massachusetts General Hospital, Harvard Medical School, Boston, MA, United States; ^2^ The Gene Lay Institute of Immunology and Inflammation, Brigham and Women’s Hospital, Mass General Hospital, and Harvard Medical School, Boston, MA, United States; ^3^ Ann Romney Center for Neurologic Diseases, Brigham and Women’s Hospital, Boston, MA, United States; ^4^ Broad Institute of Massachusetts Institute of Technology (MT) and Harvard, Cambridge, MA, United States; ^5^ Department of pharmacology and chemical biology, School of Medicine, Emory University, Atlanta, GA, United States; ^6^ Department of Microbiology and Immunology, School of Medicine, Emory University, Atlanta, GA, United States

**Keywords:** aging, immunosenescence, single cell RNA-markers, therapeutic intervention, biomarkers, T cell

## Abstract

Immunosenescence is the progressive deterioration of immune function with aging and is driven by dynamic molecular and cellular interactions, most notably the chronic low-grade inflammation (inflammaging). This inflammatory state arises from lifelong antigen exposure, environmental stress, and hormonal shifts, culminating in paradoxical immune dysfunction: innate immune cells exhibit numerical expansion but functional decline, including impaired macrophage phagocytosis and diminished dendritic cell-mediated T cell priming. Advances in single-cell RNA sequencing have uncovered biomarkers of immune aging, such as upregulation of cyclin-dependent kinase inhibitors (CDKN1A/p21 and CDKN2A/p16INK4a) and senescence-associated secretory phenotype (SASP) components like IL-6, IL-8, and TNF-α. Concurrent epigenetic dysregulation, such as EZH2-dependent H3K27me3 alterations and global DNA methylation shifts, further orchestrates immune decline. The adaptive immune system undergoes profound remodeling, marked by thymic involution, skewed T cell receptor diversity, and B cell repertoire contraction, which collectively impair responses to novel antigens and vaccination efficacy. Elucidating these mechanisms provides a roadmap for targeting strategies to restore immune resilience in aging populations.

## 1 Introduction

The world is experiencing an unprecedented rise in the number and proportion of older adults, with the population aged 60 years and over expected to increase from 1.1 billion in 2023 to 1.4 billion by 2030 and projected to reach 2.1 billion by 2050 (1, 2). This demographic shift, driven by longer life expectancy and declining fertility rates, poses significant public health challenges globally, especially in low- and middle-income countries where the change is most rapid (1).

The field of gerontology has increasingly focused on immunosenescence, the age-related decline in immune function characterized by complex alterations in both innate and adaptive immunity. This deterioration weakens host defenses, disrupts immune homeostasis, and increases susceptibility to infections, chronic inflammation, and reduced vaccine efficacy (3). Although elderly individuals exhibit increased frequencies of innate immune cells, such as macrophages and dendritic cells, their functionality is significantly impaired, which is evidenced by reduced phagocytic activity and diminished antigen presentation, which compromises T-cell priming. The observed rise in innate cell numbers is thought to reflect a compensatory response to declining immune efficiency, though this adaptation fails to restore full functionality, highlighting the multifaceted nature of immune system aging (3). Aged populations experience a higher incidence of inflammatory and degenerative diseases, such as cardiovascular disease, Alzheimer’s, and osteoarthritis, due to age-related changes in immunity (4). As the global population ages, addressing immune aging is crucial for reducing the burden of age-associated diseases and maintaining health, independence, and quality of life in older adults (5).

**Figure 1:**
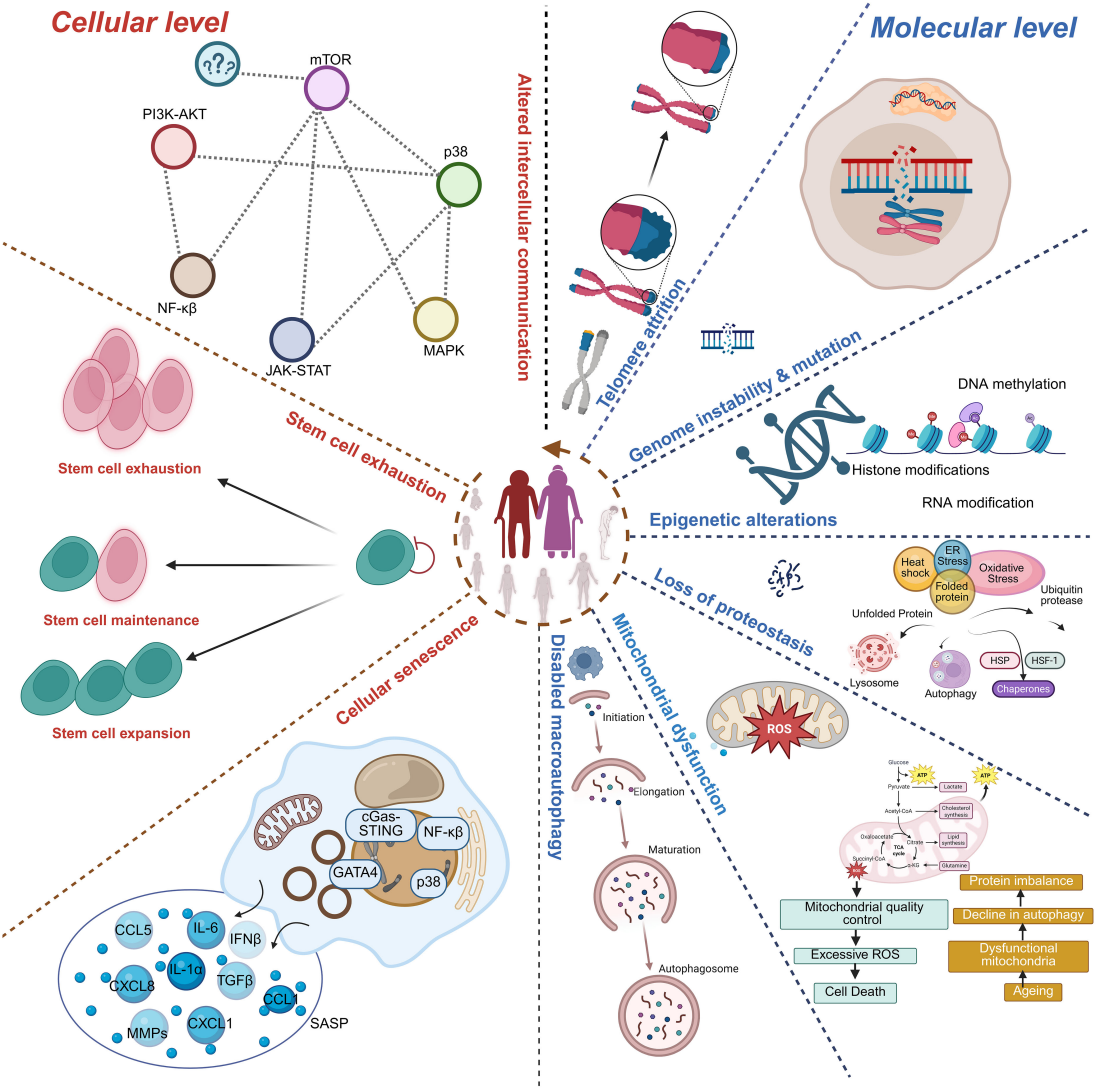
Hallmarks of immunosenescence. The schematic illustrates cellular- and molecular-level mechanisms contributing to immunosenescence. Key features include stem cell exhaustion, impaired stem cell maintenance/expansion, and cellular senescence characterized by activation of signaling pathways (PI3K-AKT, mTOR, MAPK, JAK-STAT, NF-κB, p38) and secretion of pro-inflammatory mediators (SASP). At the molecular level, immunosenescence is associated with altered intercellular communication, telomere attrition, genomic instability and mutations, epigenetic alterations (DNA methylation, histone modifications, RNA modifications), proteostasis loss, and mitochondrial dysfunction (excessive ROS, impaired autophagy, and defective mitochondrial quality control). Collectively, these hallmarks impair immune regulation and contribute to aging and age-related diseases.

A key hallmark of immunosenescence is “inflammaging,” a chronic low-grade inflammatory state fueled by persistent antigen exposure, environmental stressors, and sex hormones. Unlike acute inflammatory responses that subside post-threat clearance, inflammaging stems from dysregulated pro- and anti-inflammatory pathways. This sustained inflammation underlies multiple age-related pathologies, cardiovascular diseases, neurodegenerative disorders, and malignancies, posing significant therapeutic challenges (6). The adaptive immune system also undergoes profound alterations with aging, particularly in T-cell populations. Thymic involution, the gradual shrinkage and functional decline of the thymus, leads to a significant reduction in the production of naive T cells. This results in an accumulation of memory T cells, both antigen-experienced and naive, at the expense of naive T cells, impairing the immune system’s ability to combat novel pathogens (3). Similarly, aging and sex hormones impact B-cell function, crucial for antibody production and humoral immunity. A marked decline in B-cell repertoire diversity, reduced production of high-affinity antibodies, and impaired formation of long-lived plasma cells collectively weaken immune responses. These deficits are particularly evident in the increased susceptibility of older adults to respiratory infections such as influenza and pneumonia, as well as the reduced efficacy of vaccinations (7). Age-related declines in immune function increase vulnerability to infections, diminish vaccine effectiveness, and elevate autoimmune disorder risks in elderly populations. Deciphering these intricate mechanisms is critical for designing targeted strategies to preserve immune resilience during aging and enhance health outcomes in the elderly (**Figure 1**).

Given the profound impact of immune aging on individual and public health, there is an urgent need to unravel the underlying cellular and molecular mechanisms that drive immunosenescence and its associated pathologies. In this review, we summarized current knowledge on the hallmarks and mechanisms of immune cell aging, highlighted emerging molecular and epigenetic biomarkers, and discussed how these changes contribute to increased disease susceptibility in older adults. We further explore recent advances in single-cell technologies that provide unprecedented insights into immune cell heterogeneity during aging, and we assess promising interventional strategies aimed at rejuvenating immune function. By integrating these perspectives, we aim to provide a comprehensive framework to guide future research and therapeutic development in the field of immune aging.

## 2 Hallmarks and mechanisms of immune cell aging

**Figure 2:**
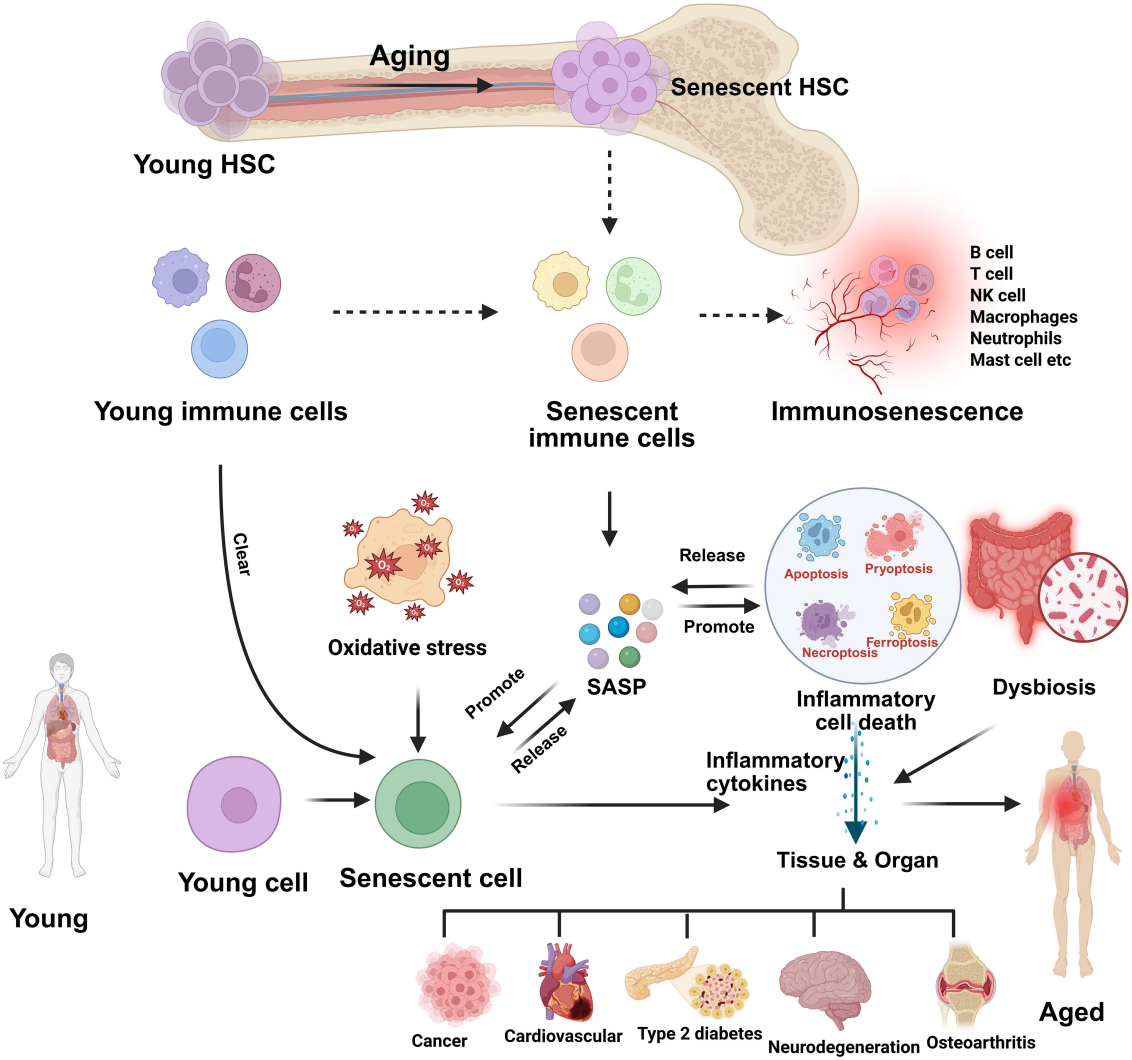
Aging-induced hematopoietic and immune senescence leading to inflammaging and tissue dysfunction. This schematic illustrates how aging drives the transition of young hematopoietic stem cells (HSCs) into senescent HSCs, which in turn generate senescent immune cells and contribute to immunosenescence. Oxidative stress and impaired clearance mechanisms promote the accumulation of senescent cells, which release a senescence-associated secretory phenotype (SASP) composed of proinflammatory cytokines, chemokines, growth factors, and proteases. SASP not only sustains chronic low-grade inflammation but also induces further senescence in neighboring cells. The release of inflammatory mediators promotes inflammatory cell death (apoptosis, pyroptosis, necroptosis, ferroptosis), dysbiosis, and tissue/organ dysfunction. Collectively, these processes culminate in age-associated systemic inflammation (inflammaging) and contribute to the onset of chronic diseases such as cancer, cardiovascular disease, type 2 diabetes, neurodegeneration, and osteoarthritis.

Immunosenescence is controlled by several key mechanisms, including cellular changes, molecular alterations, and regulatory disruptions. Cellular changes involve reduced function of both innate immune cells (such as neutrophils, macrophages, and dendritic cells) and adaptive immune cells (T and B lymphocytes) (8, 9). Molecular alterations encompass the accumulation of senescent cells with a pro-inflammatory secretory phenotype, metabolic dysregulation, and epigenetic changes (10). Additionally, senescent cells that have stopped dividing in response to damage or stress release a mix of pro-inflammatory and tissue-altering molecules known as the senescence-associated secretory phenotype (SASP). This secretory profile not only promotes inflammation but also disrupts normal tissue function, further contributing to the aging process (11). Regulatory disruptions include an imbalance in pro- and anti-inflammatory cytokines, altered immune checkpoint regulation, and impaired resolution of inflammation. These mechanisms collectively result in a decreased ability to respond to pathogens and vaccines, accumulation of memory T cells with limited diversity, increased production of pro-inflammatory mediators, and impaired clearance of senescent cells and debris. Understanding these processes is crucial for developing interventions to promote healthy immune aging and combat age-related diseases (**Table 1**; **Figure 2**).

**Table 1:** Summary of interventions used to combat ageing.

Intervention type	Strategy	Mechanism of action	Effect on immunosenescence	Reference
Pharmacological	mTOR inhibitors (e.g., rapamycin)	Inhibit mTOR pathway; promote autophagy, reduce inflammation	Enhances T cell function, improves vaccine response, and delays immune aging	(12)
	Senolytics (e.g., dasatinib + quercetin)	Selectively clear senescent cells	Reduces inflammatory milieu (SASP), improves immune tissue microenvironment	(13, 14)
	Metformin	Activates AMPK, reduces mitochondrial ROS and inflammation	Improves immune metabolism and function in aging	(15–17)
	Aspirin/NSAIDs	Reduce systemic inflammation	May lower ‘inflammaging’ and improve immune surveillance	(18)
	Hormone therapy (e.g., estrogen, DHEA)	Modulate sex hormone levels	Can restore youthful immune profiles, especially in postmenopausal women	(19, 20)
	IL-7 therapy	Cytokine that promotes T cell development and survival	Restores thymic output, enhances naive T cell pool	(21)
Lifestyle and Nutritional	Caloric restriction/Intermittent fasting	Reduces metabolic stress, promotes autophagy	Enhances immune regeneration, lowers inflammation	(22)
	Exercise (regular moderate)	Modulates cytokine profiles, enhances immune surveillance	Delays immune aging, maintains thymic mass and T cell diversity	(23)
	Dietary interventions (e.g., Mediterranean diet)	Rich in antioxidants, omega-3s, polyphenols	Reduces oxidative stress and chronic inflammation	(24)
	Supplementation (e.g., vitamin D, zinc, omega-3)	Supports immune cell function and reduces inflammation	Improves immune response, particularly in older adults	(25)
Cellular	Thymic rejuvenation (e.g., FOXN1 gene therapy)	Reverses thymic involution	Restores naive T cell output	(26)
	Stem cell therapies (e.g., HSC transplant)	Replaces aged hematopoietic stem cells	Restores immune cell generation	(27)
	Vaccination strategies (adjuvants, mRNA platforms)	Boosts immune responses tailored to aging immune systems	Improves vaccine efficacy in older populations	(28, 29)
	Microbiome modulation (probiotics/fecal transplant)	Restores gut-immune axis	Enhances mucosal immunity and systemic immune tone	(30, 31)

## 3 Structural and functional changes in lymphoid organs

### 3.1 Age-associated defects in the architecture of primary and secondary lymphoid organs

Primary lymphoid organs, including the bone marrow and thymus, play a crucial role in generating diverse immune cells. While all immune cells originate and mature in the bone marrow, T cells require additional maturation in the thymus. These organs possess a highly organized microenvironment comprising precursor immune cells and stromal cells that support immune cell development (32) However, aging leads to thymic involution, bone marrow HSC decline, and lymph node fibrosis, collectively impairing the generation and maintenance of naive immune cells and compromising adaptive immunity.

Thymic involution results in the gradual replacement of functional thymic tissue with adipose deposits, reducing naive T-cell output. In the bone marrow, hematopoietic stem cell (HSC) activity declines, impairing the body’s ability to generate new immune cells. Additionally, aging leads to a reduction in the number and size of lymph nodes, which are essential for mounting immune responses (33). In older individuals, diminished lymphadenopathy in response to infections indicates a compromised ability to activate adaptive immunity. A key structural change in aging lymph nodes is the disruption of distinct T-cell and B-cell zones due to excessive collagen deposition. This disorganization interferes with the accessibility of IL-7, a crucial cytokine necessary for T-cell survival and homeostasis. As a result, the maintenance of naive T cells is impaired, further compromising adaptive immunity (34).

### 3.2 Decreased HSC function

Aging is associated with alterations in both the frequency and function of HSCs, with variations observed across individuals based on genetic factors and underlying conditions. Studies in murine models have demonstrated that HSC frequency differs across strains, with DBA/2 mice exhibiting higher HSC counts than C57BL/6 and BALB/C strains (35). Studies have shown that the DBA/2 strain in mice exhibits a higher frequency of HSCs compared to the C57BL/6 and BALB/c strains. However, aging universally compromises HSC self-renewal and differentiation, as demonstrated by experiments transplanting aged HSCs into irradiated mice, which resulted in impaired self-renewal and reduced lymphopoiesis. A hallmark of aging is the shift in HSC differentiation toward myeloid lineages, termed skewed myelopoiesis (36). This shift is reflected in the upregulation of myeloid lineage genes over lymphoid genes, resulting in an age-related increase in myeloid cell production. Concurrently, there is a decrease in B-cell precursors, such as common lymphoid progenitors (CLPs), pro-B cells, and pre-B cells in the bone marrow, leading to diminished lymphopoiesis. Additionally, aging leads to enhanced production of reactive oxygen species (ROS), negatively impacting HSC function and further compromising immune response and hematopoietic homeostasis in the elderly population (37).

### 3.3 Age-associated TLR sensing and IFN production

Aging is also associated with reduced expression of various surface and endosomal Toll-like receptors (TLRs), which play a crucial role in the innate immune response by detecting pathogen-associated molecular patterns. This diminished expression affects the production of type I interferons (IFNα and IFNβ), key cytokines with potent antiviral effects. Type I IFNs function by inducing the expression of interferon-stimulated genes (ISGs) such as IFIT1, IFITM1, and ISG15, which are critical for inhibiting viral replication and enhancing the host’s defense mechanisms. Additionally, type I IFNs are essential in boosting antigen presentation by dendritic cells (DCs), facilitating the processing and presentation of antigens to T cells. This process promotes the expansion and activation of cognate T cells necessary for an effective adaptive immune response. The decline in type I IFN production with age weakens both the antiviral response and the overall capacity of the immune system to respond to infections and malignancies (38).

### 3.4 Aging-related alteration of innate cell response

The immune system relies on the homeostasis and functionality of innate immune cells such as DCs, macrophages, NK cells, and neutrophils. However, aging disrupts these functions, leading to compromised immune responses. This dysfunction manifests as impaired DC antigen presentation, skewed macrophage activation, accumulated yet less effective NK cells, and altered neutrophil activity (39). DCs serve as key antigen-presenting cells (APCs), bridging innate and adaptive immunity. Their primary role is to process and present antigens to T cells, initiating adaptive immune responses. In aging individuals, DC function declines due to reduced expression of costimulatory molecules like CD80 and CD86, which are essential for effective T cell activation. This impairment weakens T cell expansion and adaptive immune responses. Additionally, aging affects DC antigen uptake, processing, and migration to lymphoid tissues, leading to delayed pathogen recognition and a diminished ability to mount robust immune responses (40). Reduced efficiency in major histocompatibility complex (MHC)-mediated antigen presentation further hampers T cell recognition, contributing to increased susceptibility to infections and malignancies in older adults. Furthermore, impaired DC function diminishes vaccine efficacy, as DCs are crucial for antigen presentation and memory cell generation. This highlights the need for age-specific vaccine formulations or adjuvants to enhance DC functionality (4).

Macrophages, critical for clearing pathogens and maintaining tissue balance, undergo functional decline with aging (41). Older macrophages show a diminished ability to engulf and eliminate pathogens and cellular debris, heightening vulnerability to infections (42). Aging also shifts macrophage polarization toward the pro-inflammatory M1 state, marked by elevated secretion of TNF-α, IL-6, and IL-1β. This imbalance fosters chronic low-grade inflammation, a driver of age-associated conditions like cardiovascular diseases, type 2 diabetes, Alzheimer’s disease, and cancer. In contrast, M2 macrophages, which support tissue repair and anti-inflammatory processes, become less functional in aging, worsening impaired wound healing and unresolved inflammation (43). Aged macrophages may also develop a senescent phenotype, leading to the secretion of the SASP, which includes pro-inflammatory cytokines, chemokines, and proteases. SASP exacerbates inflammation, accelerates tissue aging, and fosters a cycle of immune dysfunction. This persistent inflammatory environment impairs immune homeostasis, increasing the risk of autoimmunity and chronic disease (44). Neutrophils, the first responders against infections, experience age-related functional impairments, including reduced phagocytosis and diminished ability to form neutrophil extracellular traps (NETs) through NETosis. These deficiencies compromise pathogen elimination, allowing infections to spread more readily in elderly individuals. Additionally, aged neutrophils exhibit prolonged inflammatory responses, contributing to tissue damage and exacerbating age-related inflammation. Chemotactic responsiveness also declines with age, reducing the efficiency of neutrophil migration to infection sites and further delaying immune responses (45). NK cells, crucial for viral defense and tumor surveillance, undergo significant changes with aging. Although their overall numbers increase, there is an accumulation of dysfunctional CD56-dim NK cells with diminished cytotoxic activity. These nonfunctional NK cells are less effective in targeting infected or malignant cells, leading to increased cancer risk and impaired viral immunity in the elderly population. The reduced cytotoxic potential of NK cells further contributes to immune senescence, as the ability to eliminate aberrant cells declines over time (46). Myeloid-derived suppressor cells (MDSCs), which regulate immune responses by inhibiting T cell activation and proliferation, accumulate excessively with age. Under normal conditions, MDSCs prevent excessive inflammation and maintain immune tolerance. However, in aged individuals, their overabundance creates an overly suppressive environment that dampens adaptive immunity. MDSCs interfere with antigen-presenting cells, release immunosuppressive molecules such as arginase, reactive oxygen species (ROS), and nitric oxide (NO), and directly inhibit T cells. This suppression not only weakens the response to infections but also reduces vaccine efficacy, as MDSCs impair T cell-mediated immunity. The excessive presence of MDSCs in older adults contributes to an overall decline in immune competence, increasing susceptibility to infections and malignancies. Targeted therapies aimed at reducing MDSC accumulation or mitigating their suppressive effects could enhance immune function in aging individuals. Recent studies on aged DC showed that Rab8a overexpression, which is accompanied by the upregulation of Rab11, restores the functionality of these aged DCs, whereas knockdown of Rab8a reduces functionality of DCs from young mice (47). Such interventions may improve T-cell responses, bolster vaccine efficacy, and enhance pathogen clearance, ultimately promoting healthier aging (48). Aging profoundly affects innate immune cell function, leading to compromised pathogen defense, chronic inflammation, and weakened vaccine responses. The decline in DC antigen presentation, macrophage phagocytic efficiency, neutrophil chemotaxis, NK cell cytotoxicity, and the excessive accumulation of MDSCs collectively contribute to immunosenescence (49). Understanding these changes is crucial for developing targeted therapeutic strategies to enhance immune resilience in older populations and mitigate age-related disease risks.

### 3.5 Effect of ageing on B cells and subtypes

As we age, our immune system undergoes significant changes, including alterations in the function and composition of B and T cells. These two primary lymphocyte populations are critical for adaptive immune responses. One notable effect of aging is that T cells experience a decline in proliferation and functionality. In contrast, B cells show impaired antibody production, contributing to an increased susceptibility to infections and a diminished response to vaccinations in older individuals. Adaptive immunity undergoes remodeling during aging, including B cell composition and function changes. A distinct subset of B cells, termed “age-associated B cells,” expands in aged mice’s spleen and bone marrow. These cells differ from conventional naive and memory B cells (6). Hao et al. identified them as CD43-CD21/CD35-CD23-B cells, while Rubtsov et al. described them as CD11b+CD11c+ B cells. Notably, these age-associated B cells express T-bet (encoded by Tbx21), similar to B cells linked to lupus-like autoimmunity in mice. Although the factors driving their expansion aren’t fully understood, evidence suggests that damage-associated molecular patterns, such as debris and chromatin from apoptotic cells, activate age-associated B cells via the TLR7 or TLR9 pathway (50, 51). Once formed, their survival in aged mice depends on IFNγ signaling. Age-associated B cells contribute to “inflammaging” by disrupting immune homeostasis, secreting IL-4 and IL-10 upon activation and presenting antigens to T cells. The recent advancements in ssRNA sequencing indicate new subsets and molecular markers for the aged B cell population like ApoE, although APOE protein is related to lipid metabolism and is expressed in other immune subpopulations. B Cells are very diverse and function in the different compartments of the body. Like splenic age-associated B cells, peritoneal B1-like cells express the transcription factor gene Zbtb32 but form a distinct transcriptional cluster. This cluster is marked by high expression of unique genes, including Zcwpw1 and Ctla4. With age, B1-like cells become more activated, strongly induce cytotoxic CD8+ T cells, and respond to commensal bacteria. Single-cell studies reveal the heterogeneity of age-associated B cells, suggesting their potential antigen specificity and tissue-specific inflammatory roles in older organisms (52). Additionally, regulatory T cells tend to increase in number with age, which may further exacerbate T cell dysfunction and contribute to a more profound immune suppression observed in elderly populations, highlighting the complex interplay between T cell dynamics and aging-related immune decline. Furthermore, this phenomenon of immune suppression due to an increased presence of regulatory T cells may be linked to the chronic inflammatory state often seen in older adults, which complicates the overall immune response and increases the risk of various age-associated morbidities, including autoimmunity and cancer (53). The role of sex hormones in modulating the aging-associated changes to the immune system has also been an area of active research. Emerging evidence suggests that sex hormones can influence the degree of immune senescence, with notable differences observed between males and females as they age, potentially leading to disparities in disease susceptibility and progression among the sexes (54).

### 3.6 Effect of ageing on T cells and subtypes

Aging significantly impacts the T cell compartment, leading to immunosenescence- a decline in naive T cell numbers that weakens responses to new infections and vaccines. This decline is largely attributed to thymic involution, where the thymus shrinks and loses functional capacity, reducing T cell progenitor output and compromising immune diversity (33). Sex differences in T cell aging suggest that males and females experience distinct immune aging patterns, likely influenced by circulating sex hormones. These differences may contribute to varying susceptibilities to age-related diseases and responses to immunization (55). Regulatory T cells (Tregs), which suppress excessive immune responses and maintain homeostasis, increase in frequency with age. While Tregs help prevent autoimmunity, their accumulation can dampen effector T cell responses, impairing the immune system’s ability to combat infections and tumors (56).

Genetic studies in mice show that premature T cell aging, such as through TFAM gene knockout- leading to systemic aging effects, affecting metabolic, musculoskeletal, cardiovascular, and cognitive health. This suggests that aging T cells may accelerate aging across multiple organ systems, making them a key target for interventions (57). Maria et al. proposed eight hallmarks of T cell aging: four primary (thymic involution, mitochondrial dysfunction, genetic/epigenetic changes, and proteostasis loss) and four secondaries (TCR repertoire reduction, naive–memory imbalance, T cell senescence, and loss of effector plasticity). These lead to two integrative hallmarks- immunodeficiency and inflammaging- characterizing aged immune function (58). Aging alters CD8+ T cells, reducing naive populations while increasing memory subsets and cloning. Single-cell RNA and TCR sequencing in mice reveal a decrease in naive CD8+ T cells across multiple tissues. A distinct PD1+TOX+CD8+ T cell subset accumulates with age, which is absent in young mice (59). Additionally, aging contributes to CD4+ T cell exhaustion, though this phenomenon is still under investigation. Using scRNA-seq-derived cell classification with our full lifespan coverage, it has been observed that although both naive T cell subsets (CD8-Naïve-LEF1 and CD4-Naïve-CCR7) exhibited a decreased trend with age (60). Understanding these age-related T cell changes is crucial for developing targeted strategies to enhance vaccine efficacy, and mitigate age-related immune dysfunction.

### 3.7 Granzyme expressing T cell subsets: key drivers and biomarkers of age-related immune remodeling

CD8+ T cells in humans demonstrate notable age-associated alterations in both phenotype and function, most evident in changes to granzyme expression. Granzymes, a family of serine proteases, are critical for cytotoxic and immunomodulatory roles. With advancing age, there is a marked expansion of granzyme K (GZMK)-expressing effector memory CD8+ T cells (TEM), identifying a subset that differs significantly from granzyme B (GZMB)-expressing TEM cells. In murine models, these GZMK+ CD8+ TEM cells, sometimes referred to as Taa (age-associated GZMK+ CD8+ T) cells, exhibit clonal proliferation and possess distinct epigenetic and transcriptional signatures associated with tissue homing, exhaustion, and a pro-inflammatory phenotype (61). Unlike their GZMB+ counterparts, GZMK+ TEM cells show reduced cytolytic activity but drive persistent low-grade inflammation by acting on non-immune cells. The accumulation of these cells is linked to increased cytokine release, recruitment of additional immune cells, and tissue damage, all of which contribute to the impaired immune landscape observed in aging.

From a mechanistic perspective, GZMK+ CD8+ TEM cells are distinguished from GZMB+ TEM cells by their capacity to induce cellular senescence in tissue fibroblasts and to intensify inflammatory niches. These cells express markers characteristic of T cell exhaustion, such as PD-1 and TOX, which closely link them to age-related immune decline. Expansion of the GZMK+ subset has been documented in several chronic conditions, including atherosclerosis, emphasizing their dual role as both indicators and potential therapeutic targets for immune aging (62). Consequently, the gradual accumulation of GZMK+ TEM cells provide crucial mechanistic insight into age-related immune remodeling and represents a promising avenue for biomarker development to track immunosenescence, persistent inflammation, and age-linked diseases (63).

Similarly, CD4+ T cells undergo age-related phenotypic shifts. The proportion of naive CD4+ T cells decline with advancing age, whereas memory and terminally differentiated subsets become more prevalent. A defining feature of senescent CD4+ T cells is the loss of CD28 expression, resulting in the emergence of CD4+CD28^null^ cells that exhibit diminished proliferative potential and natural killer (NK)-like properties, including expression of NK cell receptors and cytotoxic mediators such as perforin and GZMB (60). Although less extensively characterized, certain CD4+ T cell subsets in older individuals also upregulate GZMK, which may further amplify chronic inflammatory responses and disrupt immune regulation (64). These cytotoxic CD4+ T cells produce elevated levels of pro-inflammatory cytokines, such as IFN-γ and TNF, and preferentially accumulate in aging tissues, linking them to immunosenescence and age-associated diseases (65). Age-driven epigenetic and transcriptional adaptation supports their cytotoxic and senescent profiles, reinforcing their significance in immune aging (3).

Collectively, the expansion of GZMK+ effector memory CD8+ T cells and cytotoxic, granzyme-expressing CD4+ T cell populations positions granzymes as pivotal indicators of age-related immune remodeling. These lymphocyte subsets contribute to the perpetuation of chronic inflammation and functional deterioration, and they represent promising mechanistic biomarkers and therapeutic candidates for age-associated immune dysfunction and disease (66).

### 3.8 Effect of sex hormones on ageing immune cells

Sex hormones play a critical role in modulating age-associated changes in the immune system, contributing to distinct immune function and disease susceptibility between men and women. Estrogens, androgens, and progestins act through receptors on various immune cells, including B cells, T cells, dendritic cells, macrophages, and NK cells. Estrogens typically enhance immune responses, leading to heightened antibody production, improved B and T cell maturation, and stronger immunity in females (67, 68). Androgens, more abundant in males, generally exert immunosuppressive effects, resulting in greater susceptibility to infections but lower rates of autoimmune disorders in men. With aging, both sexes experience declines in sex hormones, but the effects differ (69). Postmenopausal women face a sharp drop in estrogen, increased inflammation (higher IL-1, IL-6, and TNF-α), and reduced immune function, contributing to “inflammaging” and a higher risk of age-related diseases. In men, declining testosterone is linked to increased inflammatory markers and changes in immune cell populations, with their adaptive immune system aging more rapidly than in women. Older women tend to have a more inflammatory immune profile but retain better adaptive immunity, explaining higher late-onset autoimmune disease rates but lower cancer incidence compared to men (70). Conversely, older men, with a more immunosuppressive hormonal environment, face greater infection and cancer susceptibility. Hormone replacement therapy in postmenopausal women can partially restore immune balance by enhancing B and T cell responses and reducing inflammation. In men, testosterone supplementation may help support immune health, whereas testosterone deficiency is linked to immune aging and is commonly treated in older populations.

SASP includes proinflammatory cytokines, chemokines, growth factors, and proteases that promote chronic inflammation and further induce senescence in neighboring cells. This cascade leads to inflammatory cell death, tissue and organ inflammation, and is influenced by alterations in the gut microbiome (71). Collectively, these processes drive age-associated systemic inflammation (inflammaging) and functional decline in aged individuals.

## 4 Molecular and epigenetic markers of immune aging

### 4.1 CDKN1A (P21)

P21 is a member of the Cip/Kip family of cyclin-dependent kinase (CDK) inhibitors, also referred to as p21 WAF1/Cip1 or CDK inhibitor 1A. Growing evidence suggests that p21^high^ represents distinct senescent subpopulations found in different tissues. It is predominantly expressed in adipocytes, callus at fracture sites, lung fibroblasts, and trophoblast cells in the placenta. It is upregulated in senescent cells and induced cell cycle arrest. scRNA-seq has identified an increased expression of CDKN1A in various aging tissues, including fibroblasts, endothelial cells, and immune cells. Cdkn1a transcript variant 2 serves as a novel and more sensitive marker of cellular senescence compared to variant 1 or total p21Cip1/Waf1 protein. It is selectively elevated during natural aging in almost all tissues of male and female mice and shows distinct temporal dynamics in response to genotoxic stress, with higher sensitivity to senolytic treatments. p21, regulated by Tet1, plays a key role in embryonic stem cell proliferation by promoting the G1 to S phase transition through the modulation of EZH2 and H3K27 trimethylation at its promoter (72). Its role in controlling cell cycle progression, cellular senescence, and tissue formation underscores its importance in both normal development and in diseases characterized by abnormal proliferation or differentiation. p21 is crucial in tissueogenesis, regulating endothelial cell proliferation during vascular growth, where Ddx21 mutation triggers Vegfc-Flt4-driven proliferation and increased p21 expression, leading to cell cycle arrest and inhibited lymph angiogenesis. p21-dependent cellular senescence is essential for neural development, where loss of Rack1 activates p21 signaling, inducing aging in neural stem cells, while removal of p21 can alleviate microcephaly caused by Rack1 knockout. It has been found that p21 maintains the viability of DNA damage-induced senescent cells by preventing DNA damage and NF-κB-mediated cell death. p21 knockout in mice reduced liver senescent stellate cells, alleviating liver fibrosis and collagen production, revealing a new pathway regulating senescent cell survival and fibrosis have reviewed many diseases such as osteoporosis, osteoarthritis, metabolic disease, chronic obstructive pulmonary disease, pulmonary fibrosis, Parkinson’s disease (PD) linked to p21 (73).

### 4.2 CDKN2A (P16INK4a)

Recent works highlight an ongoing debate regarding the reversibility of immune aging via senolytic or metabolic interventions. While the clearance of p16+ cells restore immune profiles in some models, the functional plasticity of aged cells remains limited by persistent epigenetic scars and an altered signaling landscape. P16INK4a is a well-established marker of cellular senescence and is involved in regulating the cell cycle, particularly in inhibiting CDKs. scRNA-seq has revealed that CDKN2A expression is markedly elevated in senescent cells across multiple tissues, including the liver and skin. Its expression is significantly upregulated in senescent cells and during both natural aging and age-related diseases, making p16 a key biomarker for identifying senescent cells and assessing the aging process. P16 acts as a potent inhibitor of cyclin-dependent kinases CDK4 and CDK6, preventing the G1 to S phase transition and inducing cell cycle arrest by maintaining the retinoblastoma protein (pRB) in a hypophosphorylated state (74). This regulation is thought to be part of a feedback loop, where pRB phosphorylation activates E2F, which in turn promotes p16 expression. Factors such as telomere shortening, DNA damage, and UV exposure activate the p16/pRB and p19^ARF-^p53^-^p21 signaling pathways, leading to an accumulation of senescent cells and impairing the regenerative capacity of tissues, particularly in skin stem cells. Notably, p16 expression during development coincides with the onset of cellular differentiation, a process that requires reduced cell proliferation. Consequently, p16’s role in tumor suppression aligns with its function in cell cycle inhibition. In this context, senescence in aging, marked by elevated p16 levels, can be seen as an extreme form of cell cycle arrest (75). In numerous preclinical models, clearance of senescent cells or modulation of the SASP has shown beneficial effects, with early clinical trials using senolytics yielding promising results. However, the impact of p16 ablation on health span is not straightforward, as different mouse models with p16 deletion have yielded conflicting outcomes. A comprehensive comparison of the SASP profiles across these models could provide valuable insights into how targeting specific SASP factors might improve health outcomes in aging populations. Furthermore, the high expression of p16, particularly in endothelial cells during aging, and the potential liver damage caused by the removal of p16^high^ cells warrant further investigation into the functional consequences of p16 in these cell types. A significant correlation was observed between CDKN2A mRNA expression levels and age. In Alzheimer’s disease (AD) patients, CDKN2A mRNA expression levels in blood were notably reduced, while in healthy controls, these levels increased with age. Additionally, CDKN2A mRNA expression levels in AD patients showed a significant positive correlation with DNA methylation rates (76). While CDKN1A/p21 and CDKN2A/p16INK4a are hallmarks of cellular senescence, their induction often results from upstream metabolic and signaling alterations in aged immune cells, linking mitochondrial dysfunction to epigenetic reprogramming and dysfunctional inflammatory cascades.

### 4.3 β-Galactosidase (SA-β-gal)

While not directly measured by scRNA-seq (since it’s an enzymatic activity), genes associated with SA-β-gal expression, such as GLB1, are often identified as markers for senescent cells in scRNA-seq studies. This marker is used to detect the accumulation of senescent cells in tissues. Using a second-generation fluorogenic substrate for β-galactosidase and multi-parameter flow cytometry, a study shows that peripheral blood mononuclear cells (PBMCs) from healthy humans exhibit an increasing number of cells with high senescence-associated β-galactosidase (SA-βGal) activity as donor age advances (77). The most pronounced age-related increase was observed in CD8+ T cells, with up to 64% of these cells in donors in their 60s showing high SA-βGal activity. These senescent CD8+ T cells exhibited telomere dysfunction-induced and p16-mediated senescence characteristics, including impaired proliferation, various T-cell differentiation states, and a gene expression profile similar to that seen in senescent human fibroblasts. β-galactosidase is commonly used as a tumor marker, with increased activity linked to malignant potential in cells. Oncogene activation induces oncogene-induced senescence (OIS), a process that halts the cell cycle and limits tumor cell proliferation. Initially observed in somatic non-tumor cells, OIS was later found in atypical cells (78). It leads to DNA damage, activating the p53-p21-p16 pathway, which overexpresses senescence-associated SA-βGal. This mechanism prevents atypical cells from entering mitosis, thereby halting tumor growth due to the accumulation of irreversible DNA damage. Valieva et al., reviewed SA-βGal involvement in various cancers (79).

### 4.4 SASP factors

Senescent cells exhibit distinctive molecular signatures characterized by changes in cell cycle and metabolism-related genes, along with alterations in genes encoding secretory proteins that constitute the SASP. This complex secretory program encompasses various components, including soluble signaling molecules, secreted proteases, and extracellular matrix (ECM) components, which collectively influence the tissue microenvironment (80). Through single-cell RNA sequencing analysis, researchers have identified key SASP components consistently upregulated in senescent cells. These include pro-inflammatory cytokines such as IL-6, IL-8, TNF-α, and matrix metalloproteinases (MMPs). Additionally, chemokines like CXCL1, CCL2, and growth differentiation factor 15 (GDF15) show increased expression, particularly in aging tissues. The SASP proteases are crucial in modifying the microenvironment through multiple mechanisms, including membrane protein shedding, signaling molecule degradation, and ECM modification (81). The SASP components work through interconnected pathways to maintain and amplify the senescent state. Interleukins, particularly IL-6 and IL-1, are central to this process, with IL-6 being closely linked to DNA damage-induced senescence and IL-1 activating inflammatory pathways through receptor signaling. Chemokines, including IL-8 and various MCPs, contribute to a self-reinforcing secretory network that maintains growth arrest. The insulin-like growth factor (IGF) pathway also plays a significant role, with senescent cells expressing elevated levels of IGF-binding proteins that influence senescence and apoptosis in neighboring cells (82).

Matrix metalloproteinases represent another crucial component of the SASP, with specific proteins like stromelysin-1 (MMP-3), stromelysin-2 (MMP-10), and collagenase-1 (MMP-1) being upregulated in senescent fibroblasts. These MMPs actively modify the SASP composition by cleaving various components, including MCP-1, -2, -4, and IL-8. Additionally, serine proteases, such as urokinase- and tissue-type plasminogen activators and their inhibitors, contribute to the senescent phenotype, with PAI-1 particularly important in reinforcing growth arrest (83). Beyond protein factors, senescent cells also produce non-protein molecules, including ROS and nitric oxide, which significantly impact cellular phenotypes and contribute to cancer cell aggressiveness. The influence of senescent cells extends to neighboring cell differentiation, with effects ranging from triggering epithelial-mesenchymal transition in epithelial cells to promoting angiogenesis through proangiogenic SASP components in endothelial cells. Furthermore, senescent cells actively modify the immune landscape by altering immune cell infiltration patterns, particularly in tumors, where they can recruit pro-tumorigenic T cells and macrophages (84).

### 4.5 Epigenetic markers in aging

Many studies have identified CpG sites whose methylation correlates with age; however, these epigenetic clocks are limited in their mechanistic understanding and only capture a small fraction of the genome, missing the broader context of the aging process. The researchers found that CpG sites gaining methylation with age are enriched in Polycomb Repressive Complex 2 (PRC2) targets. This age-dependent methylation gains accounts for about 90% of the increase in DNA methylation across the genome. PRC2 is primarily known for its role in transcriptional repression through the trimethylation of histone H3 at lysine 27 (H3K27me3), a marker of gene silencing. The core components of PRC2 include: (i) Enhancer of Zeste Homolog 2 (EZH2): The catalytic subunit responsible for the methylation of H3K27, which involves transferring methyl groups to the histone tail and promoting gene silencing. (ii)SUZ12: A crucial component for the stability and activity of PRC2. It is necessary to recruit the complex to chromatin and maintain H3K27me3 levels. (iii) Embryonic Ectoderm Development (EED): Binds to the methylated H3K27 mark, helping stabilize the PRC2 complex and enhancing its activity. (iv) Retinoblastoma-Binding Proteins (RBBP4/7): Co-factors that assist in chromatin binding and contribute to the overall stability of the complex. The two critical subunits, EZH2 and EED, defined below, are essential for the function of PRC2, playing a central role in regulating DNA methylation and gene silencing.

#### 4.5.1 EZh2

Aging in mice is linked to increased DNA methylation at CpG islands in regulatory regions of cancer-related genes, with conserved methylation patterns and EZH2 involvement across species, suggesting the epigenetic clock is a key feature of aging in mammals. EZH2, together with Suz12, plays a role in aging by altering the epigenome, particularly through histone modifications like H3K27me3. A recent study showed that H3K27me3 suppresses Klotho expression in the kidneys of aging mice, which is associated with aging. Histone modifications partly drive this down-regulation of Klotho at its promoter. Aging increases H3K27me3 and decreases both Klotho and mTOR hyperphosphorylation in renal tubules. Inhibiting EZH2 with drugs such as GSK343 or EED226 reduced H3K27me3 at the Klotho promoter. Despite decreased EZH2 expression in older mice, the precise role of EZH2 and H3K27me3 in renal aging is not fully understood. Some studies suggest that renal aging also up-regulates ECM laminin genes through changes in 5mC and H3K27me3. However, inhibitors like GSK-126 did not block laminin expression, highlighting the need for further investigation into EZH2’s role in renal aging (85). Emerging evidence suggests that age-related DNA methylation changes, mediated by EZH2 and other PRC2 components, potentiate NF-κB pathway activity, thereby sustaining the SASP and chronic inflammation. Thus, epigenetic drift and pro-inflammatory signaling form a self-reinforcing loop driving immunosenescence.

#### 4.5.2 EED

Human hematopoietic stem and progenitor cells (HSPCs) from umbilical cord blood have higher differentiation potential than adult HSPCs, but the molecular basis for this difference is unclear. The study identifies EED, a Polycomb Repressive Complex 2 (PRC2) member, as a key regulator. While EED expression is similar in neonatal and adult multipotent HSPCs, adult lineage-committed progenitors show higher EED levels. EED overexpression increases H3K27me3, a repressive histone modification, in adult blood cells. Genome-wide analysis reveals that adult HSPCs have higher H3K27me3 in non-hematopoietic genes, while hematopoietic genes are enriched in the active mark H3K4me3. *In vitro*, EED overexpression in neonatal HSPCs leads to abnormal myeloid marker expression, suggesting EED influences lineage decisions. These findings highlight the role of epigenetic modifications in hematopoiesis and the shift in PRC2-associated modifications from birth to adulthood. In a related study, oocyte-specific deletion of EED using Zp3-Cre recombinase in growing mouse oocytes revealed its role in oocyte programming. The deletion of EED in growing oocytes led to a significant overgrowth phenotype in offspring, including increased adiposity and bone mineral density, which persisted into adulthood. This phenotype resembles those seen in humans with Cohen-Gibson and Weaver syndromes, caused by *de novo* mutations in EED or its co-factor EZH2. The study also found that in some cases of Weaver syndrome, mutations in EZH2 occur in the maternal germline. Notably, deleting Ezh2 in mouse oocytes resulted in a distinct phenotype, indicating that EED and EZH2 have different roles in oocyte development (86).

#### 4.5.3 Bromodomain-containing protein 4

Aging is a major risk factor for vascular diseases, with recent studies highlighting the epigenetic role of BRD4 in regulating vascular pathology through transcriptional, chromatin, and DNA damage processes. BRD4 plays a key role in regulating aging-related processes and diseases, including Alzheimer’s, hypertension, and atherosclerosis, making it a promising target for therapeutic strategies. Chronic BRD4 inhibition in rat models of AD improves spatial memory by enhancing CREB signaling and synaptic protein expression, though this effect is reversed by astrocyte inhibition, highlighting the role of astrocytes in AD. Additionally, loss of BRD4 in microglial cells impairs phagocytosis and reduces the expression of AD-related genes, while combined treatment with the HDAC inhibitor MS-275 and JQ1 improves cognitive function in AD-afflicted rats. BRD4 facilitates Ang II-mediated gene expression and VSMC phenotype transition, contributing to hypertension and vascular dysfunction, with JQ1 mitigating Ang II-induced hypertension and vascular damage in mice. Clinical studies suggest that BRD4 is overexpressed in hypertension patients, and genetic mutations in BRD4 are linked to increased susceptibility to high pulse pressure, indicating that BRD4 inhibition could be a novel therapeutic approach for lowering blood pressure. BRD4 inhibition may reduce diabetic atherosclerosis by inhibiting VSMC proliferation and migration, with apabetalone preventing VSMC transdifferentiation and vascular calcification. Additionally, BRD4 regulates key processes in atherosclerosis, including macrophage function, endothelial inflammation, and apolipoprotein A-I synthesis (87).

BRD4 is essential for mammalian growth and cerebellar development, promoting cell cycle progression by regulating G1 phase gene transcription and interacting with P-TEFb and RNA polymerase II; however, both its knockout and ectopic expression can disrupt cell cycle progression through distinct mechanisms. BRD4 plays a crucial role in regulating cellular senescence by influencing telomere length and modulating p21 expression, with inhibition of BRD4 accelerating senescence in cancer cells independent of p53; however, in intervertebral disc degeneration (IVDD), BRD4 inhibition protects against senescence by activating autophagy via the AMPK/mTOR/ULK1 pathway, highlighting its complex, context-dependent effects. BRD4 also plays a complex role in cellular senescence, regulating the SASP by facilitating super-enhancer (SE) formation and promoting the expression of SASP-related genes, which can drive chronic inflammation and aging-related diseases; however, BRD4 inhibition may also offer the potential for senolysis, selectively eliminating senescent cells to alleviate aging-related conditions, highlighting the need for further research to clarify its therapeutic potential and risks. Also, BRD4 inhibition prevents LPS-induced senescence in macrophages by blocking NF-kB activation, redistributing BRD4 on chromosomes, and reducing the expression of SASP-related genes, thereby mitigating the senescent phenotype and the process of inflammaging (88).

### 4.6 Intracellular signaling pathways in aging

#### 4.6.1 PI3K/mTOR pathway

The PI3K/Akt signaling pathway regulates T cell activation, survival, metabolism, and differentiation. This pathway is activated downstream of TCR and co-stimulatory receptor signaling. In aging, the PI3K/Akt pathway becomes dysregulated. This can lead to defective T cell activation, impaired proliferation, and compromised survival of newly activated T cells. Additionally, alterations in this pathway contribute to the reduced effectiveness of the immune response to infections and tumors in the elderly. The PI3-K/Akt pathway, activated by insulin and IGF-1, plays a crucial role in aging and age-related diseases. Studies show that reducing, but not completely inhibiting, this pathway can extend a healthy lifespan in organisms from yeast to mammals. Dysregulation of PI3-K/Akt is linked to major age-related diseases, including AD. In AD, persistent activation of this pathway is associated with impaired insulin/IGF-1 responses, abnormal protein handling (Aβ and tau), synaptic loss, and cognitive decline. Targeting the PI3-K/Akt/mTOR axis to restore normal signaling has shown promise in animal models and is now being explored in clinical trials for AD and other diseases (89).

#### 4.6.2 NF-κB pathways

The NF-κB pathway is central to the inflammatory response and is activated by various signals, including those from the TCR and inflammatory cytokines. Chronic activation of NF-κB is linked to inflammation, where persistent low-grade inflammation is observed. In aging, the NF-κB pathway is often chronically activated, increasing the production of pro-inflammatory cytokines such as IL-6, TNF-α, and IL-1β. This pro-inflammatory environment exacerbates immune dysfunction, promotes T cell senescence, and contributes to the chronic inflammation seen in older individuals. NF-κB is a central regulator in aging, integrating various biochemical pathways that influence longevity. Pathways promoting aging, such as Insulin/IGF-1 signaling, mTOR, inflammation, stress, DNA damage, and telomere shortening, activate NF-κB, leading to cellular senescence, inflammation, and aging-related changes. Telomere shortening, in particular, increases NF-κB activation, creating a feedback loop that accelerates aging. In contrast, longevity factors like SIRT1, SIRT6, FOXO, and caloric restriction inhibit NF-κB signaling, promoting longevity and mitigating age-related pathologies (90).

#### 4.6.3 JAK-STAT pathway

The JAK-STAT pathway transduces signals from cytokine receptors and is crucial for immune cell differentiation, survival, and function. Cytokines like IL-2 (important for T cell proliferation) and IL-7 (important for T cell homeostasis) signal through the JAK-STAT pathway. In aging, the response to these cytokines is altered. Specifically, IL-2 signaling is often impaired in older T cells, reducing proliferative capacity. Additionally, altered IL-7 signaling impairs homeostasis and maintenance of memory T cell populations. The JAK/STAT pathway, a key regulator of inflammation, is particularly implicated in driving the SASP and has emerged as a promising therapeutic target. Inhibition of the JAK pathway, such as with ruxolitinib, has shown beneficial effects in older animals by reducing inflammation, improving metabolic function, and preserving tissue homeostasis. These findings suggest that JAK inhibitors may offer a viable treatment option for frailty and other aging-related dysfunctions, especially when combined with other therapies like metformin or rapamycin (91).

#### 4.6.4 MAPK pathway

MAPKs (Mitogen-Activated Protein Kinases) are key regulators of the NF-κB pathway, which controls the SASP. In response to senescence-inducing signals, p38 MAPK enhances NF-κB activity, promoting the transcription of proinflammatory SASP factors like IL6, IL8, and GM-CSF. Other MAPKs, including ERK1/2 and JNK, also contribute to SASP regulation by activating various substrates like MSK1, RSK1, MNK1, and MK2, which modulate protein synthesis and RNA stability, thereby enhancing the expression of SASP factors. Additionally, signaling pathways such as TGFβ and NOTCH can independently regulate specific SASP factors, further influencing the senescence response. RNA-binding proteins (RBPs) like AUF1, HuR, and ZFP36L1, regulated by MAPKs, also play crucial roles in SASP regulation by modulating the stability of SASP mRNAs (92). These MAPK-regulated mechanisms offer potential therapeutic targets for controlling SASP-related inflammation. MAPKs, including p38 and ERK1/2, are key contributors to the long-term maintenance of the senescent phenotype. Their activation increases over time in senescent cells, promoting a positive feedback loop that sustains the phenotype. For example, oxidative stress from persistent ERK1/2 activation inhibits phosphatases, while ERK1/2 and p38 promote the expression of Caveolin-1, which further inactivates phosphatases, reinforcing MAPK and p53 signaling. This signaling network supports the growth arrest characteristic of senescence. Additionally, p38 and ERK1/2 play roles in chromatin rearrangements, activation of NF-κB-STAT3 pathways, and reinforcement of mTOR signaling, all of which contribute to the stability and persistence of the senescent state. Thus, MAPKs are essential in maintaining long-term growth arrest and other features of senescence (93).

MAPKs regulate a broad array of downstream effectors, highlighting the importance of exploring their specific roles in critical senescence characteristics such as growth arrest, SASP, and resistance to apoptosis. Despite progress, many aspects of senescence remain unclear, particularly the need for more reliable and sensitive markers. Existing markers, like β-Gal activity, p21, p16, and SASP factors, lack sufficient specificity and universality. Since senescence can have beneficial and detrimental outcomes depending on the context, understanding how MAPKs influence different senescence phenotypes could lead to targeted therapeutic approaches, such as MAPK inhibitors. Moreover, there is an urgent need for improved animal models and a comprehensive catalog of the proteins and RNAs driving human senescence. Developing effective senolytic strategies to eliminate senescent cells and senostatic strategies to reprogram them will be essential. In the long term, advancing our knowledge of the signaling pathways and gene expression programs governed by MAPKs will facilitate the creation of more targeted and effective therapies (94).

## 5 Aging-associated immunological diseases

**Figure 3:**
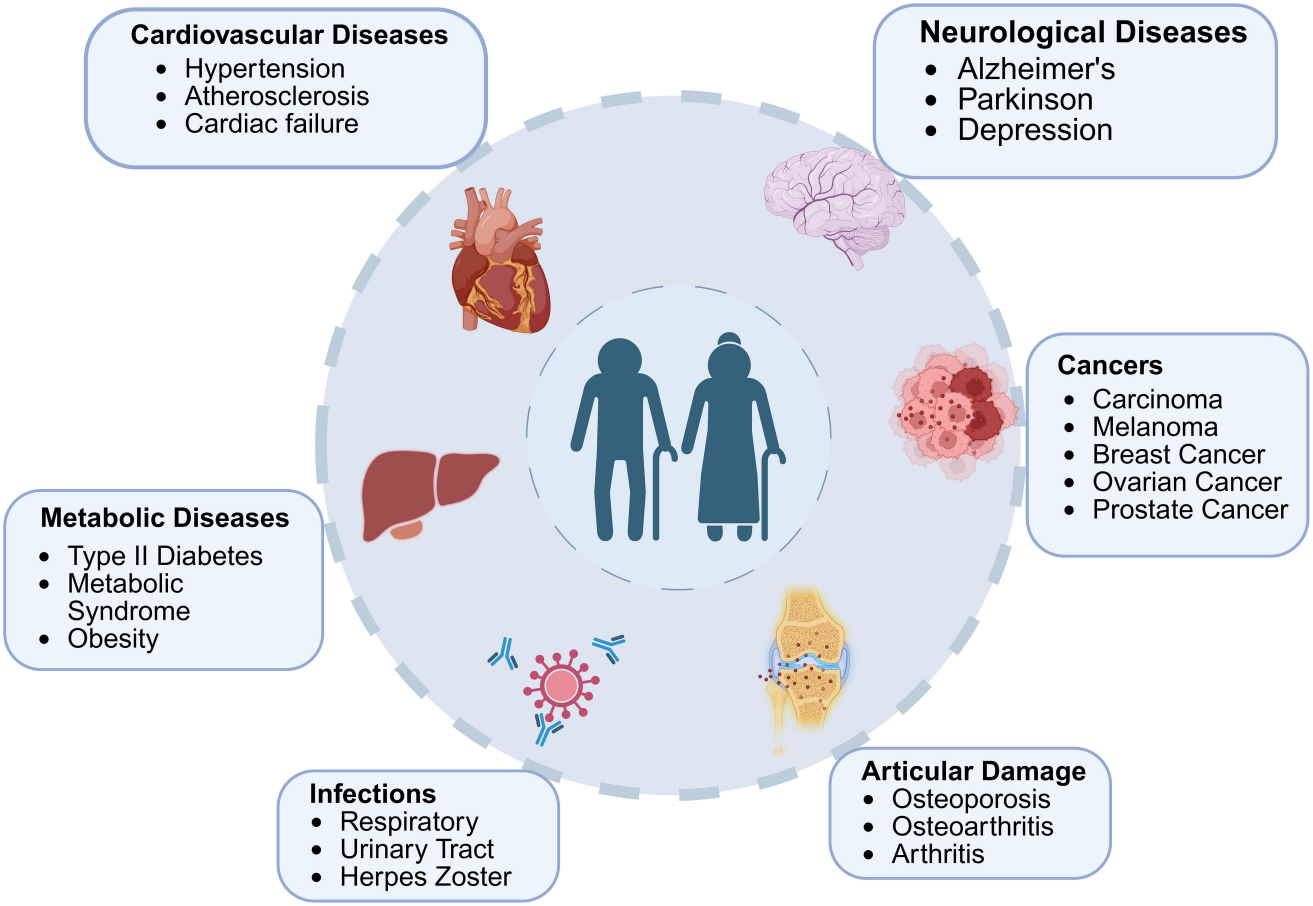
The interplay between aging and age-associated diseases. The major age-related diseases across different physiological systems, including cardiovascular, neurological, metabolic, oncological, infectious, and articular disorders is highlighted in figure. Aging contributes to the onset and progression of these conditions through shared mechanisms such as chronic inflammation, cellular senescence, and metabolic dysregulation, underscoring the interconnected nature of age-related pathologies.

Aging is closely associated with several immunological diseases due to the decline in immune function. This age-related weakening of the immune system leads to increased susceptibility to infections, poor vaccine responses, and a higher incidence of chronic inflammatory diseases, autoimmune disorders, neurodegeneration diseases, cardiovascular diseases, and cancers. Below are some key immunological diseases associated with aging **Figure 3**.

### 5.1 Increased susceptibility to infections

Immunosenescence, significantly impacts the susceptibility of older adults to various infections. Increases vulnerability to respiratory infections, urinary tract infections (UTIs), and herpes zoster (shingles) in the elderly population. Respiratory infections, particularly influenza and pneumonia, emerge as major contributors to morbidity and mortality among older adults. The compromised immune system, characterized by diminished innate and adaptive responses, struggles to combat respiratory pathogens effectively. Specifically, the reduction in T cell function and antibody production impairs the body’s ability to resist novel strains of viruses and bacteria, leaving the elderly more susceptible to these infections (95). Urinary tract infections also show increased prevalence in the aging population. This heightened occurrence stems from a combination of factors, including weakened immune surveillance, alterations in urinary tract function, and shifts in the microbiome. Notably, recurring UTIs are common among older adults, with a higher incidence observed in elderly women (96). Herpes zoster, commonly known as shingles, represents another significant health concern for older individuals. This painful condition results from the reactivation of the varicella-zoster virus, which causes chickenpox in its initial infection. The re-emergence of this latent virus is primarily attributed to immunosenescence, particularly the decline in T-cell surveillance. Consequently, older populations face an elevated risk of developing shingles (97). Understanding these age-related changes in immune function and their impact on infection susceptibility is crucial for developing targeted interventions and preventive strategies. By addressing the underlying mechanisms of immunosenescence, healthcare providers can work towards improving the quality of life and reducing infection-related complications in the elderly population (98).

### 5.2 Chronic inflammatory diseases in aging

Aging is closely linked to the onset and progression of multiple chronic conditions that compromise health and quality of life. Among these, cardiovascular disease, type 2 diabetes, and osteoarthritis represent major contributors to morbidity in older populations. Understanding the interplay between aging-related biological changes and these disorders is crucial for developing targeted interventions and preventive strategies.

#### 5.2.1 Cardiovascular disease

Chronic inflammation plays a pivotal role in the pathogenesis of cardiovascular diseases, particularly atherosclerosis. Inflammaging promotes endothelial dysfunction, oxidative stress, and vascular remodeling, leading to arterial stiffness and plaque formation. The dysregulation of immune cells, especially macrophages, contributes to a pro-inflammatory environment within the arterial walls. This environment facilitates the accumulation of lipids, autoantibodies, and autoantigens, further exacerbating the inflammatory response. The severity of inflammation correlates with an increased risk of adverse cardiovascular outcomes, highlighting the causal relationship between inflammation and cardiovascular pathology (99).

#### 5.2.2 Type 2 diabetes

The chronic inflammatory state associated with aging significantly impacts metabolic regulation, contributing to the development of type 2 diabetes. Inflammaging interferes with insulin signaling pathways, promoting insulin resistance and metabolic dysregulation. The persistent activation of inflammatory mediators, such as TNF-α and IL-6, disrupts normal glucose homeostasis and pancreatic β-cell function. This inflammatory milieu, coupled with age-related changes in adipose tissue distribution and function, creates a favorable environment for the onset and progression of type 2 diabetes in older adults (100).

#### 5.2.3 Osteoarthritis

The chronic inflammation associated with aging strongly influences Osteoarthritis, a degenerative joint disease (101). Inflammaging contributes to the breakdown of articular cartilage and alters synovial membrane function. The persistent inflammatory environment in aging joints promotes the production of matrix-degrading enzymes while inhibiting cartilage matrix synthesis. This imbalance leads to progressive joint destruction and reduced capacity for tissue repair. Combined with mechanical stress, the chronic low-grade inflammation of aging further accelerates degeneration, resulting in pain, stiffness, and reduced mobility in older individuals (102). Interestingly, inflammaging emerges as a central mechanism linking aging to the development of chronic inflammatory diseases. Understanding the underlying molecular and cellular pathways provides valuable insights into potential therapeutic targets for preventing or mitigating age-related conditions. Future research should focus on developing interventions that modulate the aging-associated inflammatory response, potentially offering new strategies to promote healthy aging and reduce the burden of chronic diseases in older populations.

#### 5.2.4 Autoimmune diseases

Immunosenescence, plays a crucial role in developing and progressing autoimmune diseases, particularly rheumatoid arthritis (RA) and giant cell arteritis (GCA). In RA, evidence suggests that premature immune system aging contributes to disease pathogenesis. T cells from RA patients exhibit features of accelerated aging, including telomere shortening and impaired DNA damage repair mechanisms. The accumulation of CD4+CD28- T cells, associated with increased production of pro-inflammatory cytokines and cytotoxicity, is a hallmark of aging and RA. GCA, an autoimmune vasculitis affecting medium and large arteries, strongly correlates with advanced age. The disease is characterized by granulomatous vessel wall inflammation, leading to Vaso occlusion and aneurysm formation. Immunosenescence contributes to GCA pathogenesis through multiple mechanisms, including dysregulation of innate and adaptive immune responses, impaired regulatory T-cell function, and age-related changes in arterial wall structure (103). Treatment strategies for both RA and GCA have advanced with the introduction of targeted biological therapies. In RA, TNF inhibitors, IL-6 receptor blockers, and B-cell–depleting agents have shown significant efficacy in reducing disease activity and slowing progression. For GCA, the IL-6 receptor blocker tocilizumab has emerged as the first approved targeted therapy, demonstrating efficacy in achieving and maintaining remission while reducing glucocorticoid use. This complex interplay between age-related immune dysfunction and autoimmunity presents both challenges and opportunities for therapeutic interventions, and deeper insights into the molecular basis of immune aging may open avenues for novel treatment approaches (104).

#### 5.2.5 Cancer

Aging is a major risk factor for cancer, with immunosenescence contributing to increased susceptibility (105). The aging immune system loses its ability to detect and eliminate cancerous cells effectively, promoting tumor growth and weakening anti-tumor defenses (33). T cells, critical for adaptive immunity, undergo significant age-related changes. Senescent T cells exhibit telomere shortening, impaired DNA repair, loss of CD28 expression, and altered metabolism. These changes reduce anti-tumor activity while increasing pro-inflammatory cytokine production, fostering an immunosuppressive tumor microenvironment. The accumulation of CD4^+^CD28^-^ T cells, a hallmark of aging and cancer, further disrupts immune balance (106).

Myeloid cells, including macrophages and dendritic cells, also experience aging-related dysfunction. Tumor-associated macrophages (TAMs) in older individuals adopt an M2-like phenotype, promoting immunosuppression and tumor progression. Aged dendritic cells exhibit reduced antigen presentation and altered cytokine production, weakening adaptive immune responses against cancer (43).

NK cells, essential for tumor surveillance, show age-related declines in cytotoxicity and cytokine production, impairing their ability to eliminate tumor cells. Additionally, regulatory T cells (Tregs) increase with age, further suppressing anti-tumor immune responses (107). At the molecular level, chronic activation of inflammatory pathways like NF-κB fosters a tumor-promoting environment. Senescent immune cells secrete pro-inflammatory cytokines and chemokines, while metabolic dysregulation and epigenetic changes further impair immune function (82).

Immunosenescence reduces the efficacy of cancer immunotherapies in older patients, necessitating tailored treatments. Strategies such as senolytic therapies, metabolic modulation (e.g., mTOR inhibition), cytokine therapies, and epigenetic modulation are being explored to counteract aging-related immune dysfunction (108).

With an aging global population, understanding and mitigating immunosenescence in cancer is crucial for improving treatment outcomes and quality of life for older adults (109). Recent preclinical studies suggest that Arf1 inhibitors induce cancer stem cell senescence and enhance anti-tumor immune responses (42). Aging drives epigenetic and transcriptional changes in mammary epithelial, stromal, and immune cells, with aging signatures also found in human breast tumors, linking aging to cancer. Among immune cells, distinct T cell subsets (Gzmk+, memory CD4+, γδ) and M2-like macrophages expand with age (110).

#### 5.2.6 Neurodegenerative diseases

The role of aging immune cells in neurodegenerative diseases with immunological components is a complex and increasingly important area of research. Immunosenescence, also contribute to the development and progression of neurodegenerative disorders such as AD and PD. This process is characterized by inflammaging, which plays a crucial role in the pathogenesis of these diseases (4).

In the context of neurodegenerative diseases, chronic inflammation contributes to several key mechanisms of disease progression. Microglial cells, the brain’s resident immune cells, become dysregulated with age, leading to impaired phagocytosis of harmful proteins like amyloid-β in AD, increased production of proinflammatory cytokines, and release of neurotoxic substances. Additionally, chronic inflammation can compromise the integrity of the blood-brain barrier, allowing peripheral immune cells to infiltrate the central nervous system and exacerbate neuroinflammation (111). In Alzheimer’s disease, aging immune cells play a particularly crucial role. Genetic factors, including mutations in microglia-associated genes, can lead to altered microglial functions such as cytokine secretion and phagocytic activity. The resulting microglial dysfunction impairs the clearance of amyloid-β peptides, leading to their accumulation and subsequent cognitive decline. The chronic state of neuroinflammation created by activated microglia further worsens neurodegeneration, creating a vicious cycle of disease progression (112). Over a century after the initial description of AD in 1906, considerable advances have been made in understanding its underlying mechanisms, refining diagnostic tools, and exploring novel therapeutic approaches (113). Contemporary research increasingly highlights the utility of integrating plasma and protein-based biomarkers including amyloid-beta (Aβ), phosphorylated tau, glial fibrillary acidic protein (GFAP), and neurofilament light chain (NfL) with genetic markers to achieve superior diagnostic accuracy (114). Among genetic risk factors, the apolipoprotein E (APOE) gene, particularly the ϵ4 allele, remains the most significant contributor to late-onset AD, exerting its influence by modulating Aβ and tau metabolism. Beyond APOE, additional genes such as ABCA7, CLU, CR1, PICALM, PLD3, TREM2, and SORL1 further shape the genetic landscape of late-onset AD (115).

Emerging evidence underscores the critical role of immune cell aging in the pathogenesis of AD, offering deeper mechanistic understanding of how immune dysfunction accelerates neurodegenerative processes. Specific alterations in gene expression including RBM3, GOLGA8A, ALS2, FSD2, and PHGDH—have been observed in aged immune and neural cells from individuals with AD, with these dysregulations correlating with disease severity and progression. For instance, reduced RBM3 expression in astrocytes compromises neuroprotective functions and cell cycle control and is also associated with decreased infiltration of CD8+ T cells, thereby representing a molecular risk factor for AD (116). In contrast, PHGDH expression is upregulated in AD, and elevated levels are linked to disease progression through effects on pathological gene regulation within the brain (117). Additionally, genes such as GOLGA8A and ALS2 are implicated in vesicle trafficking and cellular stress responses, further connecting immune dysfunction to AD-related neurodegeneration.

At the transcriptional level, a network of key regulators orchestrates the gene expression changes associated with immune cell aging in AD. For example, the transcription factor E2F1 co-regulates genes such as GOLGA8A, ALS2, RBM3, and FSD2, thereby influencing pathways involved in cell cycle progression and apoptosis (118). SPI1 (PU.1), another critical regulator, modulates ALS2, RBM3, and FSD2 expression and plays a central role in myeloid lineage immune function (118). KLF4, known for its involvement in cellular aging and immune exhaustion, is also implicated in the transcriptional remodeling underlying AD-associated immune alterations (119). Together, these regulatory factors offer mechanistic insight into how immune cell aging may drive neurodegenerative changes in AD.

Collectively, current evidence indicates that the dysregulation of genes such as RBM3, GOLGA8A, and ALS2 exerts profound effects on mitochondrial oxidative phosphorylation (OXPHOS), synaptic integrity, and immune surveillance particularly through the regulation of CD8+ T cell infiltration. These findings position immune cell aging not only as a mechanistic contributor to AD pathology, but also as a valuable source for the identification of novel biomarkers. Moving forward, research should focus on developing immune-aging biomarkers that are sensitive to the onset and progression of AD and sufficiently specific to distinguish AD from other neurodegenerative disorders. The integration of such biomarkers with established genetic and protein-based markers holds significant promise for enhancing diagnostic accuracy and guiding new therapeutic strategies (120).

PD also demonstrates significant involvement of aging immune cells in its pathogenesis. Neurohistological and neuroimaging studies support the presence of ongoing neuroinflammatory processes in PD. Alterations in peripheral blood and cerebrospinal fluid markers of inflammation and immune cell populations may initiate or exacerbate neuroinflammation. Furthermore, complex interactions between genetic predisposition and environmental factors contribute to disease development, with many disease genes and risk factors identified as modulators of immune function in PD (121).

Given the significant role of aging immune cells in neurodegenerative diseases, several therapeutic strategies are being explored to target these processes. Immunomodulatory drugs, such as NSAIDs and corticosteroids, are being studied for their potential to reduce brain inflammation. Monoclonal antibodies, like the FDA-approved Aducanumab for AD, target specific pathological proteins and modulate the immune system to reduce their accumulation. Novel immunomodulatory compounds, including immunomodulatory imide drugs (IMiDs), have shown promise in preclinical studies for various neurological diseases with inflammatory components. Additionally, drugs targeting specific inflammatory signaling pathways, such as NF-κB, JAK/STAT, and AMPK, are being investigated for their potential to regulate neuroinflammation and slow disease progression (122).

Understanding the intricate relationships between immunosenescence, chronic inflammation, and neurodegeneration is essential for developing effective therapeutic strategies. As research in this field progresses, novel approaches targeting age-related immune dysfunction may offer new hope for slowing or preventing the progression of devastating neurodegenerative disorders like AD and PD. Given the complexity of these diseases, combination therapies addressing multiple facets of immune aging and neuroinflammation may provide the most promising avenue for future treatments.

## 6 Single-cell RNA-Seq insights on aged immune cells

Single-cell RNA sequencing (scRNA-seq) has provided critical insights into immune aging by identifying key molecular markers associated with senescence and immune dysfunction (123, 124). scRNA-seq provides powerful resolution of molecular signatures and cellular diversity within aged immune systems; however, several methodological limitations must be rigorously considered to ensure accurate data interpretation. Aged tissues are particularly prone to RNA degradation, driven by increased cellular senescence, metabolic dysregulation, and elevated oxidative stress, which can compromise transcriptomic integrity and skew gene expression profiles (125). Technical variables such as extended sample processing times, inconsistent tissue dissociation methods, and reduced cell viability introduce batch effects, complicating comparative analyses across age groups or study cohorts (126, 127). Although normalization algorithms are crucial to adjust for disparities in sequencing depth and library quality, the intrinsic heterogeneity of aged populations often necessitates advanced computational strategies and meticulous experimental design to prevent artifacts and erroneous conclusions (128).

Defining and annotating cell states in aged, heterogeneous tissues presents additional challenges. Age-related increases in cellular diversity including the expansion of senescent and apoptosis-resistant populations complicate unsupervised clustering, cell-type demarcation, and lineage tracing, frequently resulting in ambiguities and reduced reproducibility across studies (125).Conventional scRNA-seq approaches lack spatial resolution, a limitation given that aged tissues display distinct microenvironmental alterations and signaling dynamics that profoundly influence immune cell phenotypes. Advances in spatial transcriptomics and integrated multi-omics hold promise for bridging these gaps, though these approaches are currently limited by high resource demands and technical complexity (60, 125).

Interpretation of scRNA-seq data from aged tissues requires careful consideration of technical noise, stochastic gene expression, and the heightened biological variability inherent to older populations. Factors such as lifetime exposures, comorbid conditions, and medication use can further confound the ability to attribute observed transcriptomic changes specifically to aging. Robust biomarker discovery and mechanistic insight will therefore depend on comprehensive study designs incorporating matched longitudinal cohorts, stringent quality control measures, and standardized analytical pipelines. Moving forward, research should emphasize methodological transparency, inclusion of adequate biological and technical replicates, and the advancement of normalization and annotation tools tailored for aging research. Meticulous reporting of methodological limitations and confounders will be critical to improving the accuracy, reproducibility, and scientific impact of single-cell studies in aged immune tissues (125, 128, 129).

**Figure 4:**
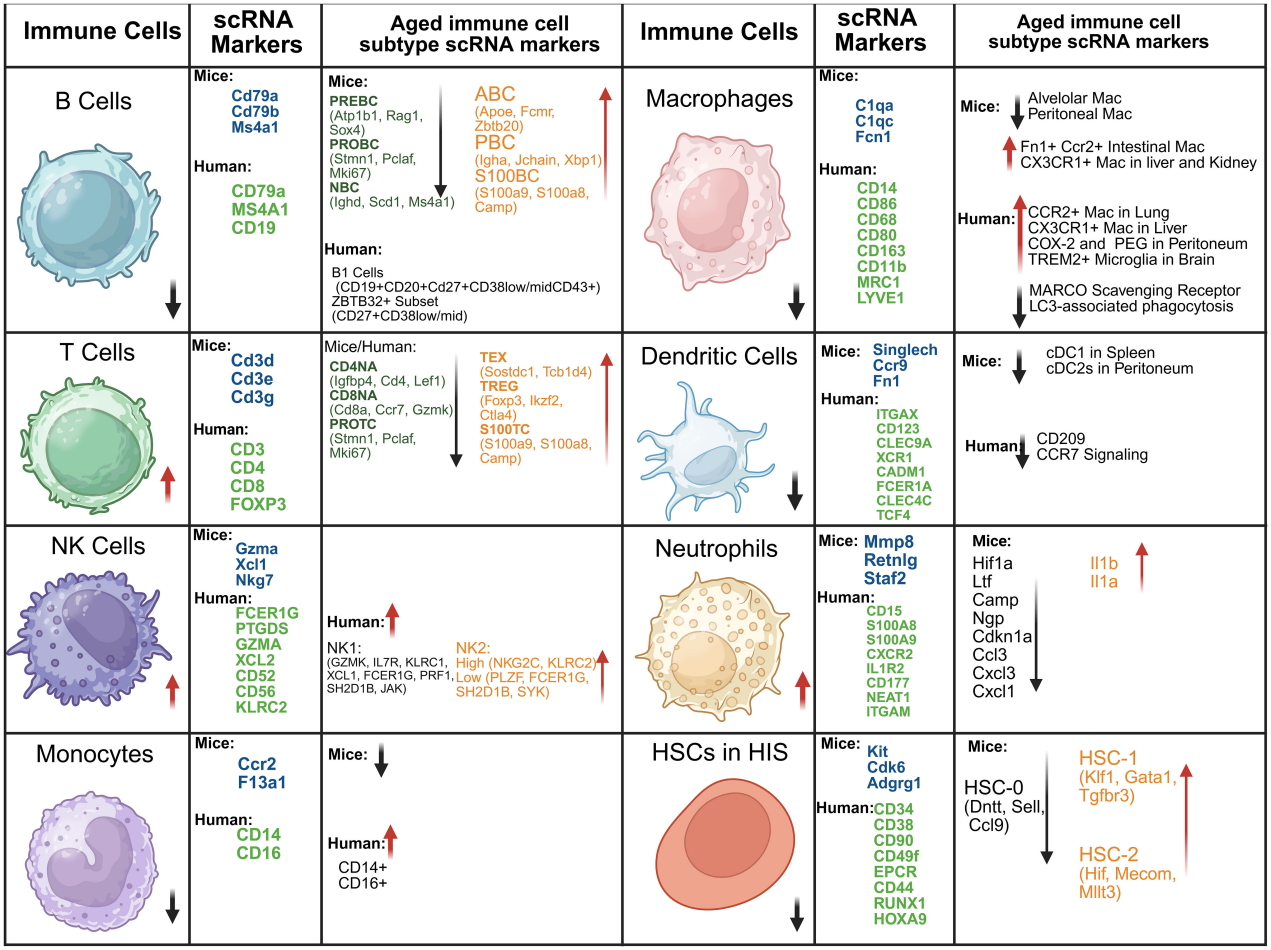
Major scRNA-sequencing markers of aging immune cells and their subpopulations in mice and humans. Key immune populations altered during aging are indicated by arrows, highlighting upregulation or downregulation. NK, Natural killer cells; DCs, Dendritic cells; HSC, Hematopoietic stem cells; BC, B cells; TC, T cells; HIS, Hematopoietic immune system; PROTC, Proliferative T cells; CD4NA, Naïve CD4+ T cells; TREG, Regulatory T cells; CD8NA, Naïve CD8+ T cells; CD8TEM, CD8+ effector memory T cells; CD8CTL, CD8+ cytotoxic T cells; S100TC, S100 T cells; TEX, Exhausted T cells; PREBC, Precursor B cells; PROBC, Progenitor B cells; NBC, Naïve B cells; S100BC, S100 B cells; PBC, Plasma cells; ABC, Age-associated B cells.

Studies in both humans and mice have revealed significant age-related changes in immune cell composition and gene expression (125). In peripheral blood mononuclear cells (PBMCs), aging is associated with increased antigen processing, chemokine signaling, and a decline in ribosomal pathway activity (125). T cells undergo notable alterations with age, including a decline in naive CD8+ T cells and an expansion of cytotoxic T lymphocytes (CTLs) exhibiting reduced CD28 expression and increased Bcl2 and Cdkn2a levels, indicating senescence and apoptosis resistance. CD4+ T cells display enhanced transcriptional heterogeneity, while B cells accumulate age-associated B cells (ABCs) marked by CD11c and T-bet expression. Super-centenarians exhibit a delayed reduction in naive and memory B cells (123). Myeloid cells, including monocytes and macrophages, show increased oxidative phosphorylation and antigen presentation activity. A shift from classical (CD14++CD16−) to pro-inflammatory non-classical (CD14+CD16++) monocytes is observed with aging (130). Natural killer (NK) cells also exhibit increased chemokine signaling gene expression, with super-centenarians demonstrating a delayed decrease in NK-GZMK cells. Senescence markers such as Cdkn1a and Cdkn2a are upregulated in aged immune cells, alongside epigenetic modifications, including global DNA methylation changes and histone modifications (125). Dysregulated microRNA expression, increased glycolysis, mitochondrial dysfunction, reactive oxygen species (ROS) accumulation, and impaired autophagy further contribute to immune senescence (131). Aging disrupts hematopoietic and immune system balance, promoting myeloid differentiation and excessive immune activation. Hematopoietic stem cells (HSCs) undergo reorganization, with reduced CD34+ and CD62L+ subsets, impairing differentiation and proliferation (132). Elevated Hif1α expression enhances neutrophil function, intensifying inflammatory responses. These findings highlight the impact of aging on immune system deterioration and underscore the need for targeted interventions to mitigate age-related immune dysfunction (133). The key single-cell markers of lymphoid and myeloid cells are summarized in **Figure 4**, which changes with aging (60, 134, 135). Although scRNA-seq has greatly expanded our understanding of immune cell heterogeneity in aging, studies of aged tissues face unique challenges. These include higher rates of RNA degradation due to increased cellular stress and senescence, pronounced batch effects from variable dissociation or handling protocols, and the complex computational task of normalizing data from highly heterogeneous cell populations. Additionally, accurately defining cell states in aged immune populations is complicated by overlapping gene expression profiles and increased transcriptional stochasticity. Future studies should incorporate robust quality control metrics, matched longitudinal sampling, and advanced normalization strategies tailored for aged tissue analyses.

## 7 Interventional strategies to mitigate immunosenescence and enhance health span

To combat immunosenescence, a range of emerging strategies aim to restore immune competence, suppress inflammaging, and extend health span. Pharmacological approaches form a key pillar of intervention (4). Senolytics and senomorphics, such as dasatinib combined with quercetin, fisetin, or BCL-2 inhibitors like navitoclax, are designed to eliminate senescent immune cells, including dysfunctional CD28− T cells, thereby reducing the pro-inflammatory SASP (136). mTOR inhibitors such as rapamycin have shown promise in enhancing T-cell memory and reducing exhaustion, extending health span in animal models (12). Metformin, through AMPK activation, exerts anti-inflammatory effects and may enhance vaccine responsiveness in the elderly (137). Hormonal modulation, including estrogen or testosterone replacement therapy, can enhance B-cell function and reduce inflammaging, though caution is warranted due to potential cancer risks (138, 139). DHEA supplementation has been explored for its potential to rebalance Th1/Th2 immunity and support thymic regeneration (140, 140).

Nutritional and metabolic interventions also show promise. Caloric restriction (CR) and fasting mimetics promote autophagy, reduce oxidative stress, and increase naïve T-cell output (141). Polyphenols such as resveratrol and curcumin suppress NF-κB-mediated inflammation and enhance dendritic cell performance. Omega-3 fatty acids can lower levels of pro-inflammatory cytokines like IL-6 and TNF-α, while boosting macrophage phagocytic activity. Adequate vitamin D3 levels are essential for correcting Th2 immune skewing and promoting antimicrobial peptide production (142). On the cellular and immunotherapy front, strategies such as thymic regeneration using IL-7 or keratinocyte growth factor (KGF) aim to replenish the declining pool of naïve T cells (143) Hematopoietic stem cell rejuvenation may help restore a balanced lymphoid-myeloid ratio. Meanwhile, CAR-T and CAR-NK cell therapies are being developed to target cancers associated with aging and potentially clear senescent cells (3).

Lifestyle and non-pharmacological interventions are equally critical. Regular aerobic and resistance exercise helps decrease the burden of senescent T cells, enhance natural killer (NK) cell function, and lower systemic IL-6 levels (3). Optimizing sleep supports melatonin-driven immune regulation and T-cell memory formation. Additionally, modulation of the gut microbiome through probiotics such as *Lactobacillus* and *Bifidobacterium* can reduce systemic inflammation and enhance mucosal immunity (144, 145). Emerging frontiers in immunosenescence research include epigenetic reprogramming. SIRT1 activators and NAD+ boosters have shown potential in improving mitochondrial function in T cells, while DNA methylation modulators may reverse age-associated epigenetic drift (146, 147). Novel vaccine strategies, including adjuvanted or mRNA-based vaccines, are being optimized to elicit stronger immune responses in older adults (28). However, several challenges remain. Personalized approaches are crucial, as sex, genetics, and baseline inflammatory status significantly influence intervention outcomes. Safety concerns persist, particularly with agents like senolytics and mTOR inhibitors, which may impair tissue repair or cause immunosuppression. Combining complementary therapies, such as rapamycin with IL-7, could yield synergistic benefits and minimize risks (3).

In conclusion, a multi-modal strategy encompassing pharmacological agents, metabolic and nutritional optimization, cell-based therapies, and lifestyle interventions offers the most promise for counteracting immunosenescence. Future clinical trials targeting specific immune cell dysfunctions and inflammatory pathways will be essential in translating these advances into effective therapies for aging populations.

## 8 Microbiota alteration in aging

The alteration of the gut microbiota has emerged as a crucial mechanistic factor and potential biomarker source in immune cell aging and related pathologies (148). The human gut microbiota, consisting of trillions of microorganisms, supports metabolic processing, immune modulation, and intestinal barrier integrity. With aging, its composition, diversity, and function undergo significant shifts commonly termed microbial dysbiosis which is linked to immunosenescence, inflammaging, and age-associated diseases (149). Longitudinal and cross-sectional studies consistently show reduced microbial diversity in older individuals, marked by loss of beneficial taxa such as *Akkermansia muciniphila*, *Bifidobacterium*, and short-chain fatty acid (SCFA)-producing bacteria, alongside expansion of opportunistic or pro-inflammatory groups like Alistipes, Bacteroides, and Proteobacteria (150). Dysbiosis disrupts gut barrier integrity, increases intestinal permeability, and promotes systemic exposure to microbial components such as lipopolysaccharide (LPS), driving persistent inflammation. In murine models, microbiota from aged donors induces gut leakiness, elevated pro-inflammatory cytokines (TNF-α, IL-6), and impaired macrophage function in young recipients, mimicking immunosenescence (151).

Conversely, colonization of aged animals with young donor microbiota restores immune homeostasis and reduces systemic inflammation, highlighting therapeutic potential.

At the molecular level, microbiota-derived SCFAs such as butyrate, acetate, and propionate reinforce epithelial barrier integrity, regulate Treg differentiation, and suppress excessive inflammation. Their decline in aging exacerbates barrier dysfunction and inflammaging (152). Microbial metabolites also modulate immune cell metabolism and epigenetic states, influencing T cell exhaustion, myeloid activation, and inflammatory balance (153). This metabolite-mediated crosstalk provides mechanistic insight into immune aging and offers avenues for biomarker discovery.

Clinically, microbiota composition and gene expression signatures correlate with physiological aging markers such as C-reactive protein, HbA1c, and cytokine profiles (154). Centenarians display distinctive microbiota enriched in anti-inflammatory taxa such as *Desulfovibrio piger* and *Gordonibacter pamelaeae*, suggesting microbial signatures of healthy aging (155). This concept of “microb-aging” positions gut microbial profiles as both indicators and modulators of biological aging (156). Beyond systemic immunity, microbiota alterations also contribute to neuroinflammation and neurodegeneration through the gut–brain axis. Dysbiosis promotes systemic inflammation, microglial activation, and blood–brain barrier dysfunction, linking microbial health to cognitive decline and Alzheimer’s pathology (157).

Therapeutically, microbiota-targeted strategies including probiotics, prebiotics, dietary modulation, and fecal microbiota transplantation (FMT) are under investigation for reversing immune aging. Clinical studies indicate that restoring microbiota balance may enhance vaccine responses, reduce gut permeability, and improve immune cell function in elderly populations, although standardization remains a challenge (158).

Recent advances in scRNA-seq reveal microbial influences on immune cell transcriptomes, showing shifts in naïve/memory T cell subsets and inflammation-associated gene expression correlating with microbial composition (154, 159). Epigenomic studies further demonstrate that microbial metabolites regulate DNA methylation and histone modifications, shaping T cell differentiation, macrophage polarization, and cytokine output (160, 161). Integrating scRNA-seq with epigenomics reveals microbiota-driven rewiring of gene regulatory networks, identifying reversible epigenetic biomarkers (160).

Advanced multi-omics and machine learning approaches that integrate microbiome, metabolome, transcriptomic, and epigenetic data promise precise biomarker panels and personalized microbiota-based therapies (157, 161).

In summary, age-associated microbiota alterations play a central role in immune cell aging and inflammaging. Their bidirectional relationship with host immunity provides mechanistic insight, biomarker opportunities, and therapeutic targets. Establishing microbiota signatures as both indicators and modifiable determinants of biological aging holds promise for promoting healthy aging and mitigating age-related diseases.

## 9 Conclusions

Understanding immunological changes in aging is critical for addressing age-related diseases and improving health outcomes in older adults. A key feature of aging immunity is “inflammaging,” a chronic low-grade inflammatory state driven by persistent antigen exposure and environmental stressors, contributing to cardiovascular, neurodegenerative, and oncological diseases. While innate immune cell numbers increase with age, their functionality declines, as seen in reduced phagocytic activity and impaired antigen presentation. Adaptive immunity undergoes significant alterations, including thymic involution, diminished naive T cell output, and reduced B cell repertoire diversity, leading to weakened immune responses. Molecular profiling through single-cell RNA sequencing has identified key aging markers, such as increased expression of senescence-associated genes (CDKN1A, CDKN2A) and pro-inflammatory SASP factors (IL-6, IL-8, TNF-α). Epigenetic modifications, including DNA methylation and histone alterations (e.g., EZH2-mediated H3K27me3), further exacerbate immune dysfunction. Dysregulation of critical signaling pathways, including PI3K/mTOR, NF-κB, JAK-STAT, and MAPK, contributes to chronic inflammation and impaired immune responses. Targeting these molecular pathways holds promise for mitigating immunosenescence and enhancing immune resilience in aging populations. Future research should focus on translating these insights into therapeutic interventions, integrating strategies that modulate inflammation, restore immune function, and improve vaccine responsiveness in older adults.

## 10 Future perspectives

**Figure 5:**
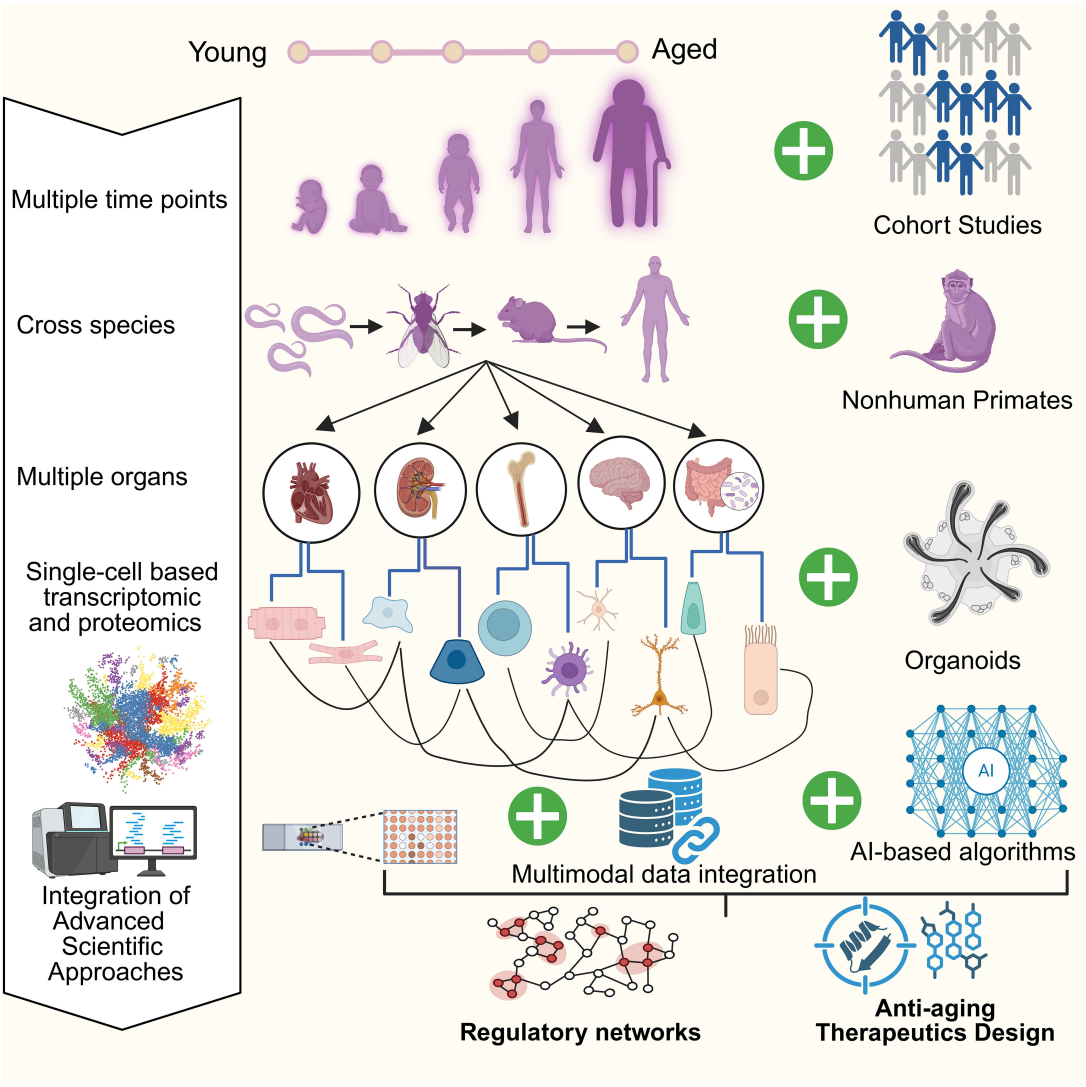
Integrative framework for aging research across species and organ systems using advanced tools. This schematic outlines a comprehensive framework for aging research that spans multiple time points, organ systems, and species. Advanced approaches such as single-cell transcriptomics and proteomics are applied to dissect cellular and molecular changes during aging. Integration of organoid platforms, nonhuman primate models, and large-scale cohort studies enhances translational relevance, while AI-based algorithms provide predictive insights and therapeutic opportunities. Multimodal data integration and regulatory network analyses form the core of this framework, supporting the rational design of anti-aging interventions. Collectively, these strategies bridge young and aged biological systems, advancing mechanistic understanding and guiding therapeutic development in aging research.

A major challenge in studying immune aging is the significant variation in aging phenotypes between mice and humans. Aging is influenced by genotype, medical history, and environment, all of which affect the immune system. Understanding these factors is essential, particularly as genetic studies have identified variability in functional decline during aging. Future single-cell and proteomics research should focus on how these factors impact different immune cell populations at various ages and how the different DNA and chromatin modifications are contributing to plasticity of immune cells. In mouse models, researchers must control for aging effects linked to other physiological changes. Moreover, analyzing the immune systems from genetically diverse individuals will uncover key mechanisms of immune adaptation to aging. Adding a temporal aspect to single-cell studies, both cross-sectional and longitudinal, is crucial to understanding the trajectories of immune aging in mice and humans. We have summarized the future approached which can be applied for better aging in **Figure 5**.
